# Relationships between changes in HIV risk perception and condom use in East Zimbabwe 2003–2013: population-based longitudinal analyses

**DOI:** 10.1186/s12889-020-08815-1

**Published:** 2020-05-24

**Authors:** Robin Schaefer, Ranjeeta Thomas, Rufurwokuda Maswera, Noah Kadzura, Constance Nyamukapa, Simon Gregson

**Affiliations:** 1 MRC Centre for Global Infectious Disease Analysis, Department of Infectious Disease Epidemiology, Imperial College London, London, UK; 2 Department of Health Policy, London School of Economics and Political Science, London, UK; 3 Biomedical Research and Training Institute, Harare, Zimbabwe

**Keywords:** HIV prevention, condom use, risk perception, Zimbabwe

## Abstract

**Background:**

Perceiving a personal risk for HIV infection is considered important for engaging in HIV prevention behaviour and often targeted in HIV prevention interventions. However, there is limited evidence for assumed causal relationships between risk perception and prevention behaviour and the degree to which change in behaviour is attributable to change in risk perception is poorly understood. This study examines longitudinal relationships between changes in HIV risk perception and in condom use and the public health importance of changing risk perception.

**Methods:**

Data on sexually active, HIV-negative adults (15–54 years) were taken from four surveys of a general-population open-cohort study in Manicaland, Zimbabwe (2003–2013). Increasing condom use between surveys was modelled in generalised estimating equations dependent on change in risk perception between surveys. Accounting for changes in other socio-demographic and behavioural factors, regression models examined the bi-directional relationship between risk perception and condom use, testing whether increasing risk perception is associated with increasing condom use and whether increasing condom use is associated with decreasing risk perception. Population attributable fractions (PAFs) were estimated.

**Results:**

One thousand, nine hundred eighty-eight males and 3715 females participated in ≥2 surveys, contributing 8426 surveys pairs. Increasing risk perception between two surveys was associated with higher odds of increasing condom use (males: adjusted odds ratio [aOR] = 1.39, 95% confidence interval [CI] = 0.85–2.28, PAF = 3.39%; females: aOR = 1.41 [1.06–1.88], PAF = 6.59%), adjusting for changes in other socio-demographic and behavioural factors. Those who decreased risk perception were also more likely to increase condom use (males: aOR = 1.76 [1.12–2.78]; females: aOR = 1.23 [0.93–1.62]) compared to those without change in risk perception.

**Conclusions:**

Results on associations between changing risk perception and increasing condom use support hypothesised effects of risk perception on condom use and effects of condom use on risk perception (down-adjusting risk perception after adopting condom use). However, low proportions of change in condom use were attributable to changing risk perception, underlining the range of factors influencing HIV prevention behaviour and the need for comprehensive approaches to HIV prevention.

## Background

In 2016, the United Nations General Assembly adopted the “Political Declaration on HIV and AIDS: On the Fast-Track to Accelerate the Fight against HIV and to End the AIDS Epidemic by 2030” (Political Declaration) that included commitments to reduce the number of new HIV infections by 75% by 2020 compared to 2010 [1]. This target will be missed by a considerable margin and the world is not on track to end the AIDS epidemic by 2030 [2–4]. In fact, in 50 countries in the world, the number of new HIV infections increased, not decreased, since 2010, including countries in sub-Saharan Africa, where 70% of global people living with HIV (PLHIV) reside and more than 60% of global new HIV infections occur [4]. This “prevention crisis” is acknowledged in the 2018 UNAIDS Global AIDS Update [4]. Similarly, the 2018 International AIDS Society-Lancet Commission details how the HIV epidemic could resurge as the largest ever generation of young people transitions into adulthood [5]. Therefore, improvements in HIV prevention efforts are needed urgently [2].

Perceiving a personal risk for HIV infection is considered important for engaging in HIV prevention behaviour [6–8]. Increasing awareness of risks and risk perception is often aimed for in HIV prevention messages and programmes in sub-Saharan Africa [9, 10]. Many HIV prevention interventions and programmes are based on theoretical assumptions of social-cognitive models of behaviour change [11, 12], and perceiving a risk for a health threat, both in terms of perceived susceptibility and perceived severity of the threat, is an important element in many of these, including the Health Belief Model [13], Social Cognitive Theory [14], Theory of Planned Behaviour [15], as well as HIV-specific theoretical models such as the AIDS Risk Reduction Model [16]. The importance attached to risk perception for HIV prevention rests on the assumption that raising risk perception has a causal effect on engaging in protective behaviour. Statistical associations have been identified between risk perception and HIV prevention behaviour, including delaying sexual debut [17] and abstinence [18, 19], condom use [20–24], and adherence to daily oral pre-exposure prophylaxis (PrEP) [25–27]. However, there is limited evidence on causal relationships between risk perception and behaviour due to lack of longitudinal studies [6, 28–30].

While causality cannot be established in observational studies, longitudinal studies can determine temporal relationships between hypothesised causes and effects, which is a prerequisite for determining causal relationships. There are few longitudinal studies on HIV risk perception conducted in sub-Saharan Africa. In a study among South African youth, individuals who started perceiving some risk for HIV infection between two surveys were more likely to get tested for HIV as an example of HIV prevention behaviour [31]. However, this effect was driven by changes in sexual risk that occurred between surveys. Moreover, for HIV testing, reverse causality is plausible, so HIV testing and counselling could cause a change in risk perception (not risk perception causing testing). In another study of the same population, males who started perceiving a risk between two surveys were more likely to report condom use during the second survey [32]. For condoms, risk perception is likely to have a stronger effect than for HIV testing and reverse causality is less likely given that starting to use condoms is implausible to cause increasing risk perception (although an increase in condom use could decrease risk perception). However, the study failed to account for condom use at baseline and included those who started to become sexually active between surveys, which the study itself found to be associated with developing risk perception.

The public health importance of changing perceptions about HIV infection risks further depends on population levels of risk perception and change in these. Even if increasing risk perception leads to increased condom use, low levels of risk perception in the population would mean that only limited numbers of people would increase condom use after increasing risk perception, so effects on population-level HIV incidence would be limited. Risk perception has often been found to be low even among those engaging in behaviours associated with increased risk of HIV infection [33–35], which may indicate that the change in prevention behaviour attributable to changes in risk perception is limited. However, again, lack of longitudinal studies limits our understanding of the degree to which behaviour can be attributed to change in risk perception. This suggests that the role of risk perception for HIV prevention is less well understood than commonly assumed.

The objectives of this study were to 1) examine longitudinal relationships between changes in HIV risk perception and condom use in a large cohort study in Zimbabwe, with the primary hypothesis being that an increase in risk perception leads to an increase in condom use and further analyses evaluating whether an increase in condom use leads to a decrease in risk perception, and 2) evaluate the public health importance of changing risk perception by estimating fractions of change in condom use attributable to change in risk perception. As opposed to previously published studies, we restricted data to those sexually active and HIV-negative to eliminate effects of sexual debut and HIV infection on condom use and risk perception. Condom use was analysed as an example of HIV prevention behaviour due limited possibilities for reverse causality, i.e. an increase in condom use is implausible to cause an increase in risk perception. Moreover, while other efficacious biomedical HIV prevention methods exist (including PrEP and voluntary medical male circumcision [VMMC]), condoms remain central to global HIV prevention efforts [4] and the importance of condom use for HIV prevention was stressed in the Political Declaration [1].

## Methods

### Setting

Data were taken from the Manicaland General-Population Cohort Study (Manicaland Study), an open-cohort study of a representative sample from three districts in Manicaland, Zimbabwe. Manicaland is a province in eastern Zimbabwe where 85% of the population live in rural areas [36]. Manicaland is characterised by an above-average proportion of individuals living under the national poverty line [37] and one of the lowest values of the human development index and life expectancy in the country [38]. In Manicaland, HIV prevalence stabilised at current levels of about 11% after a peak of over 25% in the late 1990s [39]. This is the lowest prevalence of any province in Zimbabwe, but the number of PLHIV in Manicaland is one of the highest in the country due to the large population size [40]. Behaviour change has been documented to have contributed to declines in HIV prevalence and incidence in Manicaland [41, 42]. Despite significant decreases, HIV incidence remains high at just under 1% for females and 0.5% for males in the general population [43]. Sexual relations between young women and older men, characterised by limited condom use, have been identified as a driver of new HIV infections [44]. Uptake of VMMC has been slow [45], and PrEP is only available in small-scale projects [46]. Manicaland is a priority in the Zimbabwe National HIV and AIDS Strategic Plan, with the objective of reducing HIV incidence by half by 2020 compared to 2013 [47].

### Data and measures

The Manicaland Study completed six surveys between 1998 and 2013. Data were taken from the four most recent surveys (2003–13). Study participants were selected from a household census in 12 sites (eight in the 2012/13 survey). These sites represent four different socio-economic strata of the population in Manicaland: Small towns, subsistence farming areas, agricultural estates, and roadside business centres. Members of the community acted as guides to support the implementation of the study by identifying households and members of the community eligible for the study. In the 2003–05 and 2006–08 survey, all households identified in the census were included in the study. In the 2009–11 and 2012–13 surveys, a random sample of two-thirds of households from the previous surveys in addition to a two-thirds random sample of new households identified in the census were included in the study. In the most recent survey (2012–13), four study sites were excluded (while maintaining the representation of the different socio-economic strata in the population). For the surveys included in this analysis (from the 2003–2005 to the 2012–13 survey), all individuals aged 15–54 years identified in the household census were eligible (including new in-migrants to the study sites and visitors).

Between 8000 and 15,000 adults aged 15–54 years participated in each survey, with participation rates ranging from 73.0 to 79.5%. HIV status was objectively determined for each participant on a dried blood spot sample. Other information was collected in a face-to-face interview, conducted by an interviewer of the same sex and in the local language (Shona), covering socio-demographic characteristics, sexual behaviour, perceptions and HIV-specific beliefs. Interviewers noted participants’ responses on paper questionnaires in all surveys but the most recent one (2012–13) during which data were collected electronically. To reduce social desirability bias in the reporting of sensitive information, including sexual behaviour, informal confidential interview techniques were used (allowing participants to provide information in writing rather than verbally) (see [48, 49] for details). Time between surveys was about 3 years and, among those not lost to follow-up due to out-migration or death, follow-up ranged between 77.0 and 96.4%. The Imperial College London Ethics Committee and Medical Research Council of Zimbabwe provided ethical approval for the Manicaland Study. Written informed consent was obtained from each study participant for each individual survey. A comprehensive profile of the Manicaland Study has been published elsewhere (which includes an overview of socio-demographic characteristics of the study population in each survey) [43] and more information is available online [50].

Sexually active participants who participated in at least two consecutive surveys and remained HIV-negative were included in this analysis as HIV infection impacts both risk perception and condom use. Participants who participated in non-consecutive surveys were not included given long intervals between surveys. Moreover, only participants already sexually active at first observation were included as sexual debut influences risk perception and data on condom use are only available for those sexually active. Only data from survey three of the Manicaland Study onwards (from 2003 to 2005) were included as survey measures on condom use and risk perception changed after survey two. Risk perception was measured with one survey question (“If you are not infected, do you think you are in danger of getting infected now or in the future?”), allowing for ‘yes’, ‘no’, and ‘don’t know’ responses. ‘Don’t know’ responses (9.17% of observations) were excluded from main analyses. As discussed in Additional file 1 (section 3), these excluded participants represent a diverse set of individuals that could not be easily grouped together with those perceiving or not perceiving a risk for HIV infection. Considering ‘don’t know’ as a separate category was also not meaningful as the sample was small (see Additional file 1, section 3).

Increased risk perception was defined as reporting risk perception in one survey but not in the preceding one. Decreased risk perception was not reporting risk perception in one survey but reporting risk perception in the preceding one. Condom use referred to reporting condom use during the last sexual intercourse. An increase in condom use occurred when the participant reported condom use in one survey but not in the preceding one. Further information on data and measures is provided in Additional file 1 (sections 1–2).

### Analysis

The primary hypothesis of this study was that an increase in risk perception causes an increase in condom use. However, the relationship between risk perception and condom use is bi-directional, so we further hypothesised that an increase in condom use leads to a decrease in risk perception as the protective behaviour is implemented. These hypotheses are described in Table 1. While the methods of this study only test for associations between two changes (changes in risk perception and changes in condom use) and the exact temporal relationship between these changes cannot be established (which of these changes came first), it can be tested whether the directions of associations are in theoretically expected directions (Table 1).

**Table 1:** Key hypotheses of associations between increase in condom use and change in HIV risk perception

***Among those not perceiving a risk for HIV infection at the beginning of the period between surveys:*** Hypothesis 1: An increase in HIV risk perception leads to an increase in condom use Risk perception is a motivating factor for condom use. A positive association between increased risk perception and increased condom use would support a causal role of risk perception as it is theoretically implausible that an increase in condom use causes an increase in risk perception.	
***Among those perceiving a risk for HIV infection at the beginning of the period between surveys:*** Hypothesis 2: An increase in condom use leads to a decrease in HIV risk perception Starting to use condoms may lead to a downward adjustment of risk perception as protective measures are implemented. This would be supported by a positive association between decreased risk perception and increased condom use as it would be implausible that a decrease in risk perception causes an increase in condom use.	

Further hypotheses regarding decrease in condom use are considered in Additional file 1, section 7.

Generalised estimating equation (GEE) with a logit link function for a binomial response distribution and exchangeable correlation structure was used to model changes in condom use dependent on changes in risk perception [51]. The main unit of analysis of the regressions, therefore, was a pair of two survey responses contributed by an individual. Modelling changes (*∆*) over a time period (*∆t*) removes cross-sectional interpretations of coefficients, thus making results more straightforward to interpret, and removes confounding from time-invariant unobservable factors. To model a binary outcome, the sample was restricted to those not reporting condom use at the beginning of *∆t* (*t*_−1_), so the outcome of all regressions was increase in condom use against continuing not using condoms (no change). Decreasing condom use was also considered but sample sizes were small; results are presented in Additional file 1 (section 7). GEE account for non-independence of survey responses from the same individual over time. More details on methods are provided in Additional file 1 (section 4).

Hypotheses outlined in Table 1 would only be supported by associations in expected directions in the absence of confounding by changes in other factors, although modelling changes removes the impact of time-invariant factors. For example, change in marital status is likely to impact both condom use and risk perception. It is therefore vital to account for changes in other variables associated with both risk perception and condom use. To identify variables potentially confounding the relationship between risk perception and condom use, preliminary analyses were conducted in which socio-demographic and behavioural characteristics were tested for association with both risk perception and condom use in separate logistic GEE models (see Additional file 1, section 5, for details). Following these preliminary analyses, time-variant socio-demographic and behavioural factors considered potential confounding factors included age, marital status, school enrolment, education, socio-economic status, having symptoms of sexually transmitted diseases (STDs), HIV testing, sexual risk behaviour, and having a partner who has other partners. For each of these, change between two surveys was modelled as described in Additional file 1 (section 6). Time-invariant factors cannot confound the relationship between change in risk perception and condom use as, by definition, they do not change. Sex, religious affiliation, and study site were considered time-invariant factors (very few participants reported change in religious affiliation or study site).

All GEE models, with the outcome of increase in condom use (vs. no change), included an independent variable for change in risk perception (increase/decrease vs. no change [reference category]). The change in risk perception variable can be seen to separately represent those reporting risk perception at *t*_−1_ (who may decrease risk perception) and those not reporting risk perception a *t*_−1_ (who may increase risk perception). To examine the association between change in risk perception and increase in condom use as well as whether this association may be confounded by changes in other socio-demographic and behavioural factors, models were estimated, first, including only change in age as an additional variable (model 1) and, second, including change variables for all potential confounders (model 2). Models were estimated separately by sex. In addition, these models were estimated with change in risk perception as no change against increase or decrease in risk perception broken down by reason for risk perception. These reasons refer to the reason for perceiving a risk after not reporting risk perception at *t*_−1_ or previous reason before decreasing risk perception. Reasons for risk perception included having multiple partners, having a partner who has other partners, marrying someone who may be HIV-positive, or ‘other’.

Secondary analyses estimated these models of increase in condom use in association with change in risk perception use by age group (15–24 vs. 25+ years) and marital status (not married vs. currently married) (Additional file 1, section 9). Moreover, to consider whether the relationship between change in risk perception and increase in condom use changed over time, these models were implemented by separately for different time periods between surveys (2003–2005 to 2006–2008, 2006–2008 to 2009–2011, and 2009–11 to 2012–13). Interactions were formally tested for in logistic regression models that included an interaction term of the time period and risk perception. These secondary analyses were estimated for both sexes combined (due to potential sample size limitations) and by sex. When regression models were estimated for both sexes combined, sex was included as a variable.

Population attributable fractions (PAFs) were estimated for proportions of ‘cases’ of increase in condom use attributable to increase and decrease in risk perception, respectively, as described elsewhere [52–54]. Model 2 of the regression estimates were used for this.

## Results

### Sample overview and trends in risk perception and condom use

Inclusion criteria for analysis were met by 1988 males and 3715 females, of which 38.7% participated in more than two surveys, thus contributing 4776 and 9353 survey observations, respectively, and creating 8426 survey pairs. Descriptive statistics are provided in Table 2.

**Table 2:** Socio-demographic and behavioural characteristics of the study population by HIV risk perception, Manicaland, Zimbabwe, 2003–2013

	Males (*N* = 4776)		Females (*N* = 9353)	
	Total	Risk perception	Total	Risk perception
	N (%)	N (%)	N (%)	N (%)
Age				
15–24 years	774 (16.2)	132 (17.1)	1532 (16.4)	652 (42.6)
25–54 years	4002 (83.8)	488 (12.2)	7821 (83.6)	3708 (47.4)
Marital status				
Never married	895 (18.7)	164 (18.3)	288 (3.08)	128 (44.4)
Married	3653 (76.5)	406 (11.1)	7462 (79.8)	3745 (50.2)
Separated/divorced	182 (3.81)	42 (23.1)	606 (6.48)	214 (35.3)
Widowed	46 (0.96)	8 (17.4)	997 (10.7)	273 (27.4)
School enrolment				
Not enrolled	4681 (98)	605 (12.9)	9287 (99.3)	4325 (46.6)
Currently enrolled	94 (1.97)	14 (14.9)	66 (0.71)	35 (53)
Education				
None/primary	1107 (23.2)	118 (10.7)	3801 (40.6)	1726 (45.4)
Secondary/higher	3661 (76.7)	501 (13.7)	5453 (58.3)	2595 (47.6)
Wealth index quintile				
Poorest	679 (14.2)	85 (12.5)	1434 (15.3)	667 (46.5)
2nd poorest	2126 (44.5)	271 (12.7)	4506 (48.2)	2053 (45.6)
3rd poorest	1397 (29.3)	186 (13.3)	2481 (26.5)	1202 (48.4)
4th poorest	516 (10.8)	72 (14)	806 (8.62)	396 (49.1)
Least poor	40 (0.84)	4 (10)	76 (0.81)	26 (34.2)
HIV testing in past 3 years				
No	3544 (74.2)	458 (12.9)	4789 (51.2)	2320 (48.4)
Yes	1216 (25.5)	156 (12.8)	4518 (48.3)	2015 (44.6)
STD symptoms in past 12 months				
No	4600 (96.3)	593 (12.9)	8593 (91.9)	3869 (45)
Yes	174 (3.64)	27 (15.5)	695 (7.43)	468 (67.3)
Sexual risk factors^a^				
None	3048 (63.8)	277 (9.1)	8705 (93.1)	4030 (46.3)
1	897 (18.8)	154 (17.2)	511 (5.46)	257 (50.3)
2+	797 (16.7)	186 (23.3)	78 (0.83)	44 (56.4)
Partner has other partners				
No	4577 (95.8)	576 (12.6)	7740 (82.8)	3383 (43.7)
Yes	184 (3.85)	42 (22.8)	1474 (15.8)	938 (63.6)
Condom use during last sex				
No	3812 (79.8)	434 (11.4)	8385 (89.7)	3920 (46.8)
SYes	964 (20.2)	186 (19.3)	968 (10.3)	440 (45.5)

Values are: Sample sizes (N) and relative sizes in percent (%) of the different categories of variables and, among each of these categories, the number of people and proportion perceiving a risk for HIV infection. Values may not add up to 100% due to rounding. Statistics are based on all observations (multiple observation per participant are treated as independent observations), so sample sizes are higher compared to regression analyses as unit of analysis for regressions was the survey pair. Details on measures are provided in Additional file 1, section 1.

^a^ Sexual risk factors were: Reporting more than one sexual partner in the past 12 months; reporting at least one non-regular sexual partner in the past 3 years; and reporting being in more than one sexual relationship at the moment

**Fig. 1:**
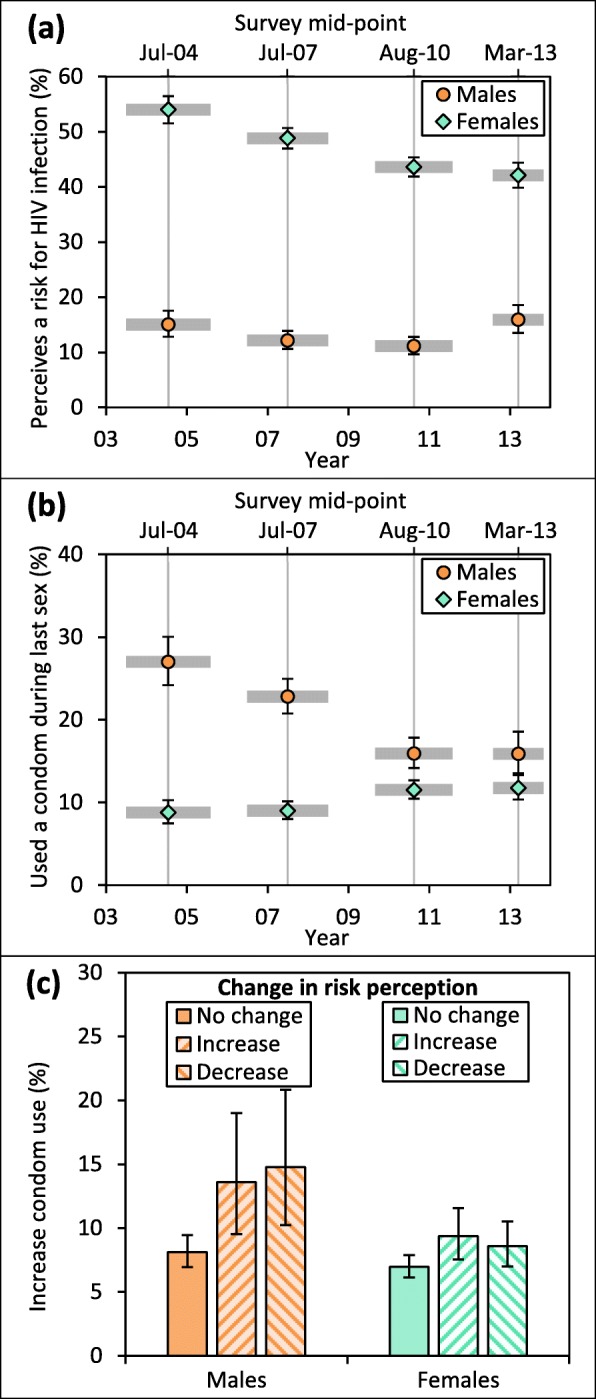
Trends in reporting of perceiving a risk for HIV infection (**a**), trends in condom use during last sex (**b**), and proportions of increase in condom use between surveys among participants with different patterns of change in perceiving a risk for HIV infection (no change; increase; decrease) (**c**), among HIV-negative, sexually active males and females (15–54 years), Manicaland, Zimbabwe, 2003–2013. Error bars indicate 95% confidence intervals. Grey areas in (**a**) and (**b**) indicate duration of surveys

Risk perception for HIV infection was reported by 13.0% of males (95% confidence interval [CI] = 12.1–14.0%) and 46.6% of females (45.6–47.6%), following declining trends over time (Fig. 1a), with an increase among males in the most recent survey. Risk perception tended to be more common among those reporting STD symptoms, sexual risk factors, and that their partners have other partners (Table 2). Reported condom use during last sexual intercourse was twice as high among males (20.2% [19.1–21.3%]) than females (10.3% [9.75–11.0%]) and increased slightly over time among females but declined markedly among males (Fig. 1b). Males reporting condom use were more likely to report risk perception than those not using condoms, but no difference in risk perception was observed among females (Table 2).

On average, 9.43% (8.40–10.6%) of males and 16.2% (15.3–17.2%) of females increased risk perception and 8.64% (7.66–9.74%) and 19.7% (18.7–20.8%), respectively, decreased risk perception between surveys. 7.21% (6.33–8.23%) of males and 6.92% (6.28–7.61%) of females increased condom use. See Table 3 for proportions of change between individual survey pairs.

**Table 3:** Changes in risk perception and condom use between surveys, Manicaland, Zimbabwe, 2003–13

	Males				Females			
	Risk perception		Condom use		Risk perception		Condom use	
Survey^a^	Increase	Decrease	Increase	Decrease^b^	Increase	Decrease	Increase	Decrease^b^
3 (2003–05) to 4 (2006–08)	8.25	10.51	7.23	13.2	14.9	18.9	6.48	5.78
4 (2006–08) to 5 (2009–11)	8.62	8.80	6.60	12.3	16.3	21.0	7.93	5.06
5 (2009–11) to 6 (2012–13)	11.8	6.40	8.00	7.02	17.1	18.9	6.06	6.28

Values are percentages (%) of change between two surveys.

^a^ Survey 1 (1998–2000) and survey 2 (2001–2003) were not included in this analysis given changing measurement of risk perception and condom use; see methods section

^b^ Decrease in condom use is considered in Additional file 1, section 7

### Increase in condom use and changes in risk perception

Proportions of males and females who increased condom use between surveys were higher among those who increased or decreased risk perception over time compared to those without change (Fig. 1c). Adjusting for other changes, increasing risk perception was associated with higher odds of increasing condom use compared to no change in risk perception among males (adjusted odds ratio [aOR] = 1.39 [0.85–2.28]) and females (aOR = 1.41 [1.06–1.88]) (Table 4) (supporting hypothesis 1). Associations were similar among younger (15–24 years) (aOR = 1.43 [0.85–2.39]) and older people (25+ years) (aOR = 1.53 [1.18–1.97]) (both sexes combined) but stronger among not married individuals (aOR = 1.86 [1.23–2.83]) compared to those currently married (aOR = 1.38 [1.02–1.87]) (both sexes combined) (Additional file 1, section 9). Decreasing risk perception was also associated with higher odds of increasing use of condoms compared to no change in risk perception among males (aOR = 1.76 [1.12–2.78]) (supporting hypothesis 2, i.e. that an increase in condom use leads to a decrease in risk perception) but showed weak association among females (aOR = 1.23 [0.93–1.62]) (model 2, Table 4). Associations were found among younger and older people and unmarried and married males but not currently married females (aOR = 1.02 [0.72–1.44]) (Additional file 1, section 9).

**Table 4:** Associations between changes in risk perception and increase in condom use between surveys, Manicaland, Zimbabwe, 2003–2013

	Males					Females				
Outcome: Increase in condom use (vs. no change)		Model 1 (*N* = 2194)		Model 2 (*N* = 2149)			Model 1 (*N* = 5084)		Model 2 (*N* = 4832)	
Variable	n (%)	aOR	(95% CI)	aOR	(95% CI)	n (%)	aOR	(95% CI)	aOR	(95% CI)
Change in risk perception										
No change in risk perception	1812 (82.6)	1 (Reference)		1 (Reference)		3173 (64.4)	1 (Reference)		1 (Reference)	
No risk perception → Risk perception (increase)^a^	206 (9.39)	1.79	(1.16–2.75)	1.39	(0.85–2.28)	822 (16.2)	1.42	(1.08–1.85)	1.41	(1.06–1.88)
Risk perception → No risk perception (decrease)^a^	176 (8.02)	1.91	(1.22–2.98)	1.76	(1.12–2.78)	989 (19.5)	1.23	(0.95–1.60)	1.23	(0.93–1.62)
Change in risk perception with reason^b^										
No change in risk perception	1812 (82.9)	1 (Reference)		1 (Reference)		3273 (64.8)	1 (Reference)		1 (Reference)	
No risk perception → Risk perception (reason)										
Has multiple partners	28 (1.29)	3.78	(1.58–9.08)	3.12	(1.12–8.72)	7 (0.14)	NA^c^		NA^c^	
Partner has other partners	29 (1.34)	0.94	(0.23–3.82)	0.37	(0.04–3.29)	229 (4.53)	1.70	(1.10–2.63)	1.57	(0.98–2.51)
Marry HIV-positive partner	25 (1.15)	9.30	(4.14–20.9)	6.93	(2.5–19.25)	25 (0.49)	7.10	(2.90–17.4)	5.37	(1.93–14.9)
Other	120 (5.54)	0.62	(0.27–1.44)	0.55	(0.23–1.36)	551 (10.9)	1.12	(0.80–1.58)	1.18	(0.82–1.69)
Risk perception (reason) → No risk perception										
Has multiple partners	23 (1.05)	6.55	(2.74–15.7)	6.73	(2.64–17.2)	14 (0.28)	1.04	(0.14–8.00)	NA^c^	
Partner has other partners	39 (1.78)	1.09	(0.37–3.22)	0.97	(0.34–2.76)	313 (6.19)	1.60	(1.10–2.35)	1.57	(1.03–2.38)
Marry HIV-positive partner	20 (0.91)	2.99	(1.13–7.94)	2.39	(0.86–6.61)	28 (0.55)	10.1	(4.66–21.7)	7.72	(3.47–17.2)
Other	89 (4.07)	1.32	(0.66–2.65)	1.31	(0.65–2.61)	613 (12.1)	0.84	(0.58–1.20)	0.89	(0.61–1.29)

Values are: Sample sizes (n) and percentages (%) for changes in risk perception; sample sizes for regression models (N); and adjusted odds ratios (aOR) with 95% confidence intervals (95% CI). Sample: Sexually active, HIV-negative participants (15–54 years) not reporting condom use at the beginning of periods between surveys. Outcome of regressions: Increase in condom use vs. no change (continuing not using condoms). Increased and decreased risk perception was compared to no change (risk perception or no risk perception in both surveys). Estimates for other independent variables are not shown. Sample sizes differ between models due to missing data on included variables.

Model 1: Change variables included for: Age group.

Model 2: Change variables included for: Age group, marital status, educational attainment, school enrolment status, socio-economic status, STD symptoms, sexual risk, partner concurrency, HIV testing (lifetime), HIV testing (past three years).

^a^ A positive association between an increase in risk perception and the outcome (increase in condom use) would support hypothesis 1 (an increase in risk perception leads to an increase in condom use). A positive relationship between a decrease in risk perception and the outcome would support hypothesis 2 (an increase in condom use leads to a decrease in risk perception)

^b^ Reasons for risk perception refer to the reasons given at the end of the period between surveys for increasing risk perception or at the beginning for decreasing risk perception

^c^ No association of change in risk perception in this category with change in condom use could be estimated

Among males, associations of increased and decreased risk perception with increased condom use were particularly strong among those perceiving a risk due to having multiple partners and because they may marry an HIV-positive partner (Table 4). Among females, similarly, associations were strongest among those perceiving a risk for HIV infection because they may marry an HIV-infected partner but, as opposed to males, also strong among those reporting risk perception because their partners have other partners (Table 4).

The majority of those who increased condom use did so without changing risk perception (Table 5). Among males, 201 ‘cases’ of increased condom use between surveys were observed. Of these, 28 reported increased risk perception, with an estimated PAF of 3.39% (− 2.22–8.70%), and 26 reported decreased risk perception (PAF = 5.29% [0.34–9.99%]). Among females, 390 ‘cases’ of increased condom use were observed, of which 77 reported increased risk perception (PAF = 6.59% [0.54–12.3%]) and 85 decreased risk perception (PAF = 4.63% [− 1.96–10.8%]).

**Table 5:** Population attributable fractions for increase in condoms due to changes in risk perception, Manicaland, Zimbabwe, 2003–13

	Increase in condom use					
	Males			Females		
	n/N (%)	PAF	(95% CI)	n/N (%)	PAF	(95% CI)
Increased risk perception	28/201 (13.9)	3.39%	(−2.22–8.70%)	77/390 (19.7)	6.59%	(0.54–12.3%)
Decreased risk perception	26/201 (12.9)	5.29%	(0.34–9.99%)	85/390 (21.8)	4.63%	(−1.96–10.8%)

Values are: Number of people who increased or decreased risk perception (n) and their percentage (%) among everyone who increased condom use (N); and population attributable fraction (PAF) and 95% confidence interval (95% CI), indicating the proportion of increase in condom use due to the change in risk perception. These estimates are based on adjusted odds ratios (model 2 estimates in Table 4).

### Increase in condom use and changes in risk perception in different time periods

In all time periods, an increase in risk perception was associated with higher odds of increasing condom use compared to not changing risk perception (Table 6). However, adjusting for other changes (model 2), for both sexes combined, the strength of this association decreased over time (2003–2005 to 2006–2008: aOR = 1.78 [1.10–2.87]; 2006–2008 to 2009–2011: aOR = 1.41 [0.97–2.04]; 2009–2011 to 2012–2013: aOR = 1.18 [0.74–1.89]). There were no significant interactions (overall interaction in model 2: *p*-value = 0.408) (not shown). Similarly, for males and females separately, the association was strongest in the earliest time period (2003–2005 to 2006–2008) (male aOR = 2.05 [0.72–5.82]; female aOR = 1.72 [0.98–3.02]) (Table 6, model 2). For both sexes combined, decreasing risk perception was associated with increased odds of increasing condom use in all time periods, but there were no clear patterns (Table 6). For males, there was a strong interaction in the earliest time period (2003–2005 to 2006–2008) (aOR = 2.63 [1.12–6.16]) and the most recent time period (2009–2011 to 2012–2013) (aOR = 2.10 [0.89–4.97]). For females, there was only an association in the intermediate time period (2006–2008 to 2009–2011) (aOR = 1.66 [1.12–2.46]). No interaction (either both sexes combined or by sex) was statistically significant (all interaction terms in regression models and overall interactions: p-value> 0.10) (not shown).

**Table 6:** Associations between changes in risk perception and increase in condom use between surveys at different time periods, Manicaland, Zimbabwe, 2003–2013

	Both sexes combined					Males					Females				
Outcome: Increase in condom use		Model 1		Model 2			Model 1		Model 2			Model 1		Model 2	
Variable	n (%)	aOR	(95% CI)	aOR	(95% CI)	n (%)	aOR	(95% CI)	aOR	(95% CI)	n (%)	aOR	(95% CI)	aOR	(95% CI)
**Period between 2003 and 2005 and 2006–2008**															
Change in risk perception															
No change	1485 (71.3)	1 (Reference)		1 (Reference)		532 (82.4)	1 (Reference)		1 (Reference)		953 (66.4)	1 (Reference)		1 (Reference)	
Increase	263 (12.6)	1.89	(1.22–2.93)	1.78	(1.10–2.87)	53 (8.20)	2.65	(1.19–5.90)	2.05	(0.72–5.82)	210 (14.6)	1.71	(1.02–2.86)	1.72	(0.98–3.02)
Decrease	334 (16.0)	1.36	(0.88–2.10)	1.23	(0.78–1.94)	61 (9.44)	2.62	(1.22–5.63)	2.63	(1.12–6.16)	273 (19.0)	1.02	(0.59–1.75)	0.99	(0.56–1.75)
**Period between 2006 and 2008 and 2009–2011**															
Change in risk perception															
No change	1999 (69.1)	1 (Reference)		1 (Reference)		713 (83.2)	1 (Reference)		1 (Reference)		1286 (63.2)	1 (Reference)		1 (Reference)	
Increase	404 (14.0)	1.42	(0.99–2.03)	1.41	(0.97–2.04)	73 (8.52)	1.30	(0.60–2.83)	1.15	(0.49–2.70)	331 (16.3)	1.50	(1.00–2.25)	1.50	(0.98–2.29)
Decrease	490 (16.9)	1.38	(0.98–1.95)	1.49	(1.06–2.10)	71 (8.28)	1.12	(0.46–2.71)	1.00	(0.36–2.79)	419 (20.6)	1.44	(0.99–2.09)	1.66	(1.12–2.46)
**Period between 2009 and 2011 and 2012–2013**															
Change in risk perception															
No change	1601 (69.5)	1 (Reference)		1 (Reference)		567 (82.1)	1 (Reference)		1 (Reference)		1034 (64.1)	1 (Reference)		1 (Reference)	
Increase	361 (15.7)	1.31	(0.86–1.99)	1.18	(0.74–1.89)	80 (11.6)	1.65	(0.78–3.49)	1.27	(0.55–2.94)	281 (17.4)	1.14	(0.68–1.92)	1.13	(0.63–2.04)
Decrease	341 (14.8)	1.33	(0.85–2.06)	1.18	(0.73–1.91)	44 (6.37)	2.21	(0.94–5.20)	2.10	(0.89–4.97)	297 (18.4)	1.07	(0.65–1.77)	0.98	(0.56–1.70)

Values are: Sample sizes (n) and percentages (%) for changes in risk perception and adjusted odds ratios (aOR) with 95% confidence intervals (95% CI). Sample: Sexually active, HIV-negative participants (15–54 years) not reporting condom use at the beginning of periods between surveys. Outcome of regressions: Increase in condom use vs. no change (continuing not using condoms). Increased and decreased risk perception was compared to no change (risk perception or no risk perception in both surveys). Estimates for other independent variables are not shown. Regressions were implemented for both sexes combined and by sex for the different time periods.

Model 1: Change variables included for: Age group.

Model 2: Change variables included for: Age group, marital status, educational attainment, school enrolment status, socio-economic status, STD symptoms, sexual risk, partner concurrency, HIV testing (lifetime), HIV testing (past three years).

## Discussion

Using 10 years of longitudinal data from a general-population cohort in east Zimbabwe, we find support for hypothesised causal links between risk perception for HIV infection and condom use. Males and females who increased risk perception between surveys were more likely to increase condom use. Although observational studies cannot determine causality, establishing the temporal relationship between cause and effect is a prerequisite for establishing causal relationships. As it is not plausible that condom use causes risk perception, the association between increased risk perception and increased condom use found in this study supports the hypothesis that risk perception causes condom use (hypothesis 1). Similarly, those who decreased risk perception between surveys were more likely to increase condom use. As it is not plausible that decreased risk perception causes condom use, this supports the hypothesis that there is a circular feedback between risk perception and condom use, so those who implemented the protective behaviour (condom use) adjust their risk perception downward (hypothesis 2). As opposed to a previous study in South Africa that similarly concluded that increased risk perception leads to condom use [32], influences of sexual debut and HIV infection were eliminated by restricting analyses to those HIV-negative and sexually active, and changes in other socio-demographic and behavioural factors that may confound associations between risk perception and condom use were controlled for.

As found by another study on the study population in Manicaland [55], HIV risk perception was low, particularly among males, including those reporting potential sexual risk factors. Moreover, while associations between changes in risk perception and condom use support a causal relationship, fractions of change in condom use in the population attributable to changes in risk perception were small. Less than 4% of increased condom use among males and 7% among females could be attributed to increased risk perception. Most change in condom use occurred without any measured change in risk perception, and the majority of those changing risk perception did not change condom use behaviour. This may be partly because condoms are also used for family planning purposes, so condom use decisions are influenced by non-HIV-related motivations; however, condoms play a marginal role in family planning in Zimbabwe compared to other modern contraceptives [56]. More importantly, low proportions of condom use change attributable to change in risk perception underscore the range of factors that influence HIV prevention behaviour. Even within social-cognitive models of behaviour change, risk perception is only one among several factors, including perceived benefits and barriers as well as self-efficacy. In fact, a meta-analysis of social-cognitive model constructs in relation to condom use in sub-Saharan Africa found associations with risk perception to be weaker compared to perceived consequences of condom use and self-efficacy [30].

While interactions between various cognitive concepts focused on in social-cognitive models are increasingly integrated in theoretical models [57], there are also various factors determining HIV prevention behaviour that may be beyond the individual’s control [58, 59] – as has been recognised for a broad range of health behaviours [60]. For condom use, particularly, partner refusal may represent a barrier to using condoms in situations where one partner lacks negotiation power, as may be the case in sexual relations between young women and older men – although we found similar effects of increased risk perception on increased condom use among males and females and younger and older females. The importance of partner-related factors is also illustrated by the fact that increased risk perception because the individual may marry an HIV-infected partner was strongly associated with increased condom use. Awareness of the partner’s positive HIV status may be a strong determinant of perceiving a risk for HIV infection and there may be fewer barriers to condom use as it may be more acceptable for couples to use condoms compared to situations where partners are (or believe to be) concordant HIV-negative. In this regard, this study underscores potential benefits of couples-based HIV testing and counselling [61]. Moreover, factors relating to friends and peers, the family, and the community as well as structural factors may represent facilitators or barriers to HIV prevention use over which individuals have little control. This is increasingly recognised in approaches to HIV prevention [3, 62]. A review of HIV prevention interventions among South African youth, for instance, found that, while most interventions are based on social-cognitive models, they also tend to address one or more social or structural barrier to HIV prevention [63]. Such approaches include addressing gender inequalities through economic empowerment [64] or promotion of positive gender norms [65, 66] and community mobilisation [67]. The role of policy and legal barriers to HIV prevention is also increasingly acknowledged to be central to the HIV response [3].

HIV risk perception has been declining over time and, for both sexes, the association between increase in risk perception and increase in condom use has become weaker over time, suggesting that less increase in condom use could be attributed to increase in risk perception in the more recent time periods. In a previous analysis of the study population [55], the accuracy of risk perception in terms of its association with actual HIV acquisition has been found to be declining over time. This may suggest that the importance of perceiving a risk for HIV infection on its own for engaging in HIV prevention behaviour has been declining, possibly due to general decreases in motivation to engage in HIV prevention behaviour or increases in other barriers to engaging in prevention behaviour, although it is important to note that there is large uncertainty around estimates for different time periods due to smaller sample sizes and interactions were not statistically significant.

### Limitations

Other individual or non-individual factors that may have influenced changes in condom use were not analysed, so no inference can be made regarding the relative importance of other factors in this context. There may also be unobserved confounding by other factors not considered in this analysis, although a broad range of factors were considered and included in regression models, which are likely to also at least partially capture changes in other factors not included in models. Another limitation was that long intervals between surveys may have limited the ability to capture change in condom use and risk perception, which may change within short time periods, thus possibly underestimating fractions of condom use change attributable to changing risk perception. Low proportions of change between surveys in risk perception and condom use, however, suggest that these may be relatively stable characteristics, so changes may still be adequately captured. Moreover, reporting bias may have led to underreporting of condom use and risk perception (as evident in different levels of reporting of condom use between males and females, which may, in part, reflect different patterns of reporting bias), despite interview methods to reduce bias [48, 49]. This may affect regressions and PAFs in various ways, but it seems unlikely that PAFs would be strongly increased with more accurate measurement of risk perception and condom use, particularly among females as levels of risk perception were high.

There are also limitations in the measures used for condom use and risk perception. Condom use during last sexual intercourse may not reflect condom use over longer periods. Nevertheless, previous analyses of this study population [68] and other studies [69, 70] found condom use during last sexual intercourse to be a good indicator for condom use during the past 2 weeks. The risk perception measure in this study captured perceived susceptibility, not perceived severity of HIV infection, although perceived severity may significantly impact behaviour, so more change in behaviour may have been attributed to risk perception if more aspects of risk perception were captured. Furthermore, relationships between risk perception and condom use may differ between regular and non-regular partners. Associations between increased risk perception and increased condom use were stronger among unmarried people and particularly strong among males increasing risk perception due to having multiple partners, suggesting that effects of risk perception due to sexual relations with non-regular partners may be stronger, but interactions by relationship type could not be explored.

## Conclusions

To meet international targets of reducing numbers of new HIV infections and prevent a resurgence of the HIV epidemic, strengthening HIV prevention efforts is needed urgently. With results likely to be generalisable to other parts of sub-Saharan Africa [71], this study documents longitudinal associations that support causal links between risk perception and using condoms, thus supporting HIV prevention interventions aiming at raising risk perceptions, but small population attributable fractions underscore the need for comprehensive approaches to HIV prevention, avoiding a narrow focus on perceptions about HIV infection risks. This applies to all HIV prevention methods. Although the partner may play a particularly strong role for condom use, partner support has been found to be important for high adherence to PrEP among sub-Saharan African women [72] and uptake of VMMC [73]. Creating conducive social environments for using HIV prevention methods is similarly crucial [62], and the drive towards community-owned HIV prevention programmes is central to this [3]. As for many health behaviours, it is therefore vital to acknowledge the myriad of factors that influence the use of HIV prevention methods, acting and interacting at different levels, and to move beyond a focus on biomedical prevention tools in isolation, ignoring social and structural drivers of HIV epidemics [74–76].

## Supplementary information

**Additional file 1.** One document (.pdf) containing the following sections, referred to throughout the article: 1. Further information on data and measures (p.2). 2. Information on the imputation procedures (p.3). 3. Analysis of excluded responses for the risk perception measure (p.4). 4. Details on methods (p.5). 5. Preliminary analyses to identify potential confounding factors (p.6). 6. Description of modelling change for potential confounding factors (p.7). 7. Analysis of decrease in condom use in relation to change in risk perception (p.8). 8. Sensitivity analysis of different definition of ‘no change’ in risk perception (p.13). 9. Additional results of analyses by age groups and marital status (p.15)

## Data Availability

Data produced by the Manicaland Project can be obtained from the project website: http://www.manicalandhivproject.org/data.html. Here we provide a core dataset which contains a sample of socio-demographic, sexual behaviour and HIV testing variables from all 6 surveys of the main survey. If further data is required, a data request form must be completed (available to download from our website) and submitted to simon.gregson@imperial.ac.uk.

## Abbreviations

AIDS: Acquired immunodeficiency syndrome
CI: Confidence interval
GEE: Generalised estimating eq.
HIV: Human immunodeficiency virus
aOR: Adjusted odds ratio
PAF: Population attributable fraction
PLHIV: People living with HIV
PrEP: Pre-exposure prophylaxis
STD: Sexually transmitted disease
VMMC: Voluntary medical male circumcision
