# Supplementary material for: Relationships between changes in HIV risk perception and condom use in East Zimbabwe 2003–2013: population-based longitudinal analyses
Source: BMC Public Health. 2020 May 24;20:756. doi: 10.1186/s12889-020-08815-1 (PMC7245904; doi:10.1186/s12889-020-08815-1)
Supplement: Supplementary file 1 — Additional file 1. One document (.pdf) containing the following sections, referred to throughout the article: 1. Further information on data and measures (p.2). 2. Information on the imputation procedures (p.3). 3. Analysis of excluded responses for the risk perception measure (p.4). 4. Details on methods (p.5). 5. Preliminary analyses to identify potential confounding factors (p.6). 6. Description of modelling change for potential confounding factors (p.7). 7. Analysis of decrease in condom use in relation to change in risk perception (p.8). 8. Sensitivity analysis of different definition of ‘no change’ in risk perception (p.13). 9. Additional results of analyses by age groups and marital status (p.15) [file 12889_2020_8815_MOESM1_ESM.docx]

**Supplementary Material**

**Relationships between changes in HIV risk perception and condom use in east Zimbabwe 2003-2013: Population-based longitudinal analyses**

**Schaefer et al.**

**BMC Public Health**

**This supplementary material contains:**

1. Further information on data and measures (p.2)
2. Information on the imputation procedures (p.3)
3. Analysis of excluded responses for the risk perception measure (p.4)
4. Details on methods (p.5)
5. Preliminary analyses to identify potential confounding factors (p.6)
6. Description of modelling change for potential confounding factors (p.7)
7. Analysis of decrease in condom use in relation to change in risk perception (p.8)
8. Sensitivity analysis of different definition of ‘no change’ in risk perception (p.13)
9. Additional results of analyses by age groups and marital status (p.15)

## Additional information on data and variables

Descriptions of the data underlying variables of this study are provided below.

**Risk perception**

- *Risk perception:* From survey round 3 (2003-2005), risk perception was based on answers to the question “If you are not infected, do you think you are in danger of getting infected now or in the future?” with “yes”, “no”, and “don’t know” response possibilities. In the two surveys before 2003, risk perception was measured with “Do you think you could become infected with HIV yourself in the future?” with the same response possibilities. Participants responding with “don’t know” where excluded, as discussed further below.
- *Risk perception reasons:* Reasons for perceiving a risk for HIV infection were determined with the question “Why do you think you might become infected”. The response categories “regular partner had many partners” and “future partner may have other partners” were grouped together.

**Condom use**

- *Condom use:* Condom use was based on the question “Did you use condoms throughout the last time you have sex?” that was asked from survey round 3 (2003-5). During earlier survey rounds condom use was measured only for the past two weeks, which is not directly comparable.

**Socio-demographic and other characteristics**

- *Education:* As there very few individuals with no or with higher education, two categories of educational attainment were created: ‘no or primary education’ and ‘secondary or higher education’.
- *Marital status:* Marriage was defined as a long-term relationships that lasted for at least 12 months.
- *Socio-economic status:* A wealth index variable was created to represent socio-economic status. This was based on characteristics of and items present in the household, so it does not directly measure individual wealth. The index was based on sellable assets like cars and non-sellable assets like the water source; this index ranged from zero to one and was divided into quintiles.
- *HIV testing:* HIV testing was measured with the question “On how many different occasions have you had an HIV test: (i) in your lifetime; (ii) in the last 3 years?”; the reporting of lifetime HIV testing was used to create a ‘ever HIV test’ variable, while the reporting of HIV testing in the past three years was used to create a ‘recent HIV test’ variable.
- *STD symptoms:* The STD symptoms variable was based on reporting of noticing any of the following symptoms in the past 12 months: discharge from the penis (males), discharge from the vagina (females), pain during urination (males), pain in the lower stomach (females), sores in the genital area (both sexes).

**Sexual behaviour**

- *Sexual risk factors:* The sexual risk factor variable used in the study was an index based on the number of positive responses to the three separate variables of multiple sexual partners, casual sexual partners, and concurrent sexual partners.
- *Multiple sexual partners:* Reporting more than one sexual partner for the question “How many different sexual partners have you had in the last 12 months?”
- *Casual sexual partners:* Reporting at least one non-regular partner for the question “How many different non-regular sexual partners have you had in the last three years?”
- *Concurrent sexual partnerships:* Reporting more than one sexual relationship for the question “How many sexual relationships do you consider yourself to be involved in at the moment?”
- *Partner concurrency:* Partner concurrency was based on whether the respondent reported at least one other partner for the question “If you took a guess, how many partners other than yourself (and any co-wives) do you think your current spouse/partner has had in the last 12 months?”

## Imputation of information for missing data

The longitudinal nature of the data was used to impute information for some variables included in the regression analyses in the main article if these had missing information. About 1% of the data used in the main regression analyses had information imputed for at least one variable. This was imputed using the data from previous or following survey, if data were available in any of these surveys. The exact imputation procedure varied depending on the variable, as outlined in Table S1. For the imputation of data, data from earlier surveys excluded in the main analyses (survey round two) were also used (for example marital status at survey round two may be used if a participant was missing data on this in survey round three). The data were imputed before excluding participants not eligible for the main analyses. For instance, a participant may have become HIV-infected between surveys four and five, so data from survey five would be excluded from the main analyses, but data from survey round five may be imputed for missing data at round four. However, data were never imputed for participants with gaps in survey participation, e.g. missing data in round five would not be imputed from round three if the participant did not participate in round four. In the data used in the main analyses, 165 observations were affected by these imputations.

| **Table S1:** Imputation of data for main analyses. | | |
| --- | --- | --- |
| **Variable** | **Imputation procedure for missing data at time t** | **N** |
| HIV status | If individuals were HIV-negative at t+1, this was imputed for t. If individuals were HIV-positive at t-1, this was imputed for t. This imputation was implemented even if there were gaps in survey participants for participants. | 48 |
| Ever sexual intercourse | If individuals reported having never had sexual intercourse at t+1, this was imputed for t. If individuals reported having had sexually intercourse at t-1, this was imputed for t. This imputation was implemented even if there were gaps in survey participants for participants. | 0 |
| Age | If missing data for age at time t, the age at t+1 minus three years or the age at t-1 plus three years was imputed. | 0 |
| Marital status | First, when reporting having never been married at time t+1, this was imputed for t. After this, marital status at t-1 or t+1 was imputed for marital status at t. This assumes that marital status is relatively constant over time. | 55 |
| Education | If individuals reported no or primary education at t+1, this was imputed for t. If individuals reported secondary or higher education at t-1, this was imputed for t. | 25 |
| School enrolment | If individuals reported being enrolled in school at t+1, this was imputed for t. If individuals reported not being enrolled in school at t-1, this was imputed for t. | 15 |
| Wealth index quintile | Missing data for the wealth index quintile was imputed from t-1 or t+1, assuming that socio-economic status is relatively stable over time. | 25 |
| HIV testing (lifetime) | If participants reported having never done an HIV test at t+1, this was imputed for t. If participants reported having done an HIV test before at t-1, this was imputed for t. This imputation was implemented even if there were gaps in survey participants for participants. | 20 |
| The table describes the method used for imputing information missing for a variable at one survey (time t) from the subsequent survey (t+1) or previous survey (t-1). The number of study participants for which data was imputed for each variable is indicated (N). This refers to participants included in the main analyses of the study. | | |

## Analyses of excluded responses for risk perception

Participants responding “don’t know” to the question risk perception were excluded from all analyses. This includes 205 males (4.00% of male sample) and 1301 females (11.4%). As outlined in Table S2, proportions for many socio-demographic and behavioural characteristics for those responding “don’t know” are between the proportions of those perceiving and those not perceiving a risk. This underscores that this group includes a diverse set of individuals that could not easily be grouped together with either those perceiving a risk or those not perceiving a risk. Moreover, including “don’t know” as a separate category was not meaningful as the sample was small.

| **Table S2:** Socio-demographic and behavioural characteristics in the study sample by risk perception response and sex, Manicaland, Zimbabwe, 2003-2013. | | | | | | | | |
| --- | --- | --- | --- | --- | --- | --- | --- | --- |
|  | | **Male** | | |  | **Females** | | |
|  | | **Does not perceive risk (N=4272)** | **Perceives risk (N=645)** | **Don’t know (N=205)** |  | **Does not perceive risk (N=5403)** | **Perceives risk (N=4707)** | **Don’t know (N=1301)** |
| Age | |  |  |  |  |  |  |  |
|  | 15-24 years | 15.3 | 21.4 | 11.7 |  | 17.8 | 14.9 | 16.6 |
|  | 25-54 years | 84.7 | 78.6 | 88.3 |  | 82.2 | 85.1 | 83.4 |
| Marital status | |  |  |  |  |  |  |  |
|  | Never married | 17.7 | 27.0 | 18.5 |  | 3.24 | 2.87 | 3.07 |
|  | Married | 78.0 | 64.8 | 73.7 |  | 74.4 | 85.7 | 80.9 |
|  | Separated/divorced | 3.42 | 6.98 | 7.32 |  | 7.79 | 5.10 | 7.76 |
|  | Widowed | 0.91 | 1.24 | 0.49 |  | 14.6 | 6.31 | 8.30 |
| Education | |  |  |  |  |  |  |  |
|  | None/primary | 24.0 | 19.3 | 24.0 |  | 42.5 | 39.7 | 46.1 |
|  | Secondary/higher | 76.0 | 80.8 | 76.0 |  | 57.5 | 60.3 | 53.9 |
| Wealth index quintile | |  |  |  |  |  |  |  |
|  | Poorest | 14.3 | 13.5 | 12.8 |  | 15.5 | 15.5 | 13.9 |
|  | 2^nd^ poorest | 45.2 | 43.9 | 45.6 |  | 49.6 | 47.2 | 47.8 |
|  | 3^rd^ poorest | 29.2 | 30.2 | 28.9 |  | 25.8 | 27.9 | 29.8 |
|  | 4^th^ poorest | 10.5 | 11.8 | 10.3 |  | 8.09 | 8.83 | 7.71 |
|  | Least poor | 0.85 | 0.62 | 2.45 |  | 0.97 | 0.58 | 0.77 |
| Sexual risk factors | |  |  |  |  |  |  |  |
|  | None | 69.5 | 50.5 | 69.0 |  | 94.4 | 93.5 | 94.3 |
|  | 1 | 19.8 | 29.3 | 16.3 |  | 5.11 | 5.83 | 4.82 |
|  | 2+ | 10.8 | 20.1 | 14.7 |  | 0.47 | 0.69 | 0.86 |
| Partner has other partners | |  |  |  |  |  |  |  |
|  | No | 96.5 | 92.9 | 95.2 |  | 89.1 | 78.7 | 87.9 |
|  | Yes | 3.53 | 7.13 | 4.88 |  | 10.9 | 21.3 | 12.1 |
| Condom use during last sex | |  |  |  |  |  |  |  |
|  | No | 81.3 | 69.8 | 77.6 |  | 89.8 | 90.1 | 91.1 |
|  | Yes | 18.7 | 30.2 | 22.4 |  | 10.2 | 9.94 | 8.92 |
| Values are percentages of the overall sample for males and females. Values may not add up to 100% due rounding. | | | | | | | | |

## Details on methods

Data from one participant over several surveys are not independent. This correlation in the data is accounted for in generalised estimating equations (GEEs). GEEs produce marginal (population average) models and produce robust parameter estimates even if the correlation structure is not correctly identified.^1^ GEEs are estimated with a logit link function for a binomial response distribution.

Coefficient estimates in GEE models have both cross-sectional (between-subject) and longitudinal (within-subject) interpretations. For example, in a model of condom use (outcome) and risk perception (predictor), an odds ratio (OR) of 1.40 can be interpreted as 1) subjects reporting risk perception have, on average, 40% higher odds of reporting condom use, and 2) subjects who develop risk perception over time have, on average, 40% higher odds of starting to use condoms over time. It is therefore vital to differentiate between cross-sectional and longitudinal effects.

To remove cross-sectional effects, instead of values of variables at one point in time, changes ($\Delta$) are modelled:

|  | $\left( Y_{it}-Y_{it-1} \right)=\Delta Y_{i\Delta t}=\beta_{0}+\sum_{j=1}^{J} \beta_{j}\left( X_{ijt}-X_{ijt-1} \right)+\ldots=\beta_{0}+\sum_{j=1}^{J} \beta_{j}\Delta X_{ij\Delta t}+\ldots$ | (1) |
| --- | --- | --- |

Where $Y_{it}$ is the observation for individual i at time t, $Y_{it-1}$ is the observation for individual i at time t-1, and $\Delta Y_{i\Delta t}$ is the change in Y for individual i over the time period $\Delta$t; $\beta_{0}$ is the intercept, $X_{ijt}$ is the independent variable j for subject i at time t, $X_{ijt-1}$ is the independent variable j for individual i at time t-1, and $\Delta X_{ij\Delta t}$ is the change in variable j in individual i over time period $\Delta$t; $\beta_{j}$ is regression coefficient for the independent variable j, and J is the number of independent variables.

To model changes in binary outcomes using GEE, models must be run separately for different starting values of the outcome variable as a change variable for a binary outcome has four categories. In this study, models are estimated separately for those reporting and not reporting condom use at t-1. This is a disadvantage over autoregressive (Markov) models, which model outcomes at a current time t while controlling for the outcome at previous times. However, modelling changes completely removes cross-sectional effects, removing confounding from time-invariant unobservable factors, and so results are more straightforward to interpret.

GEE requires defining a correlation structure that determines the impact of data from the same individual from past surveys. When modelling changes, the correction for within-subject correlation is part of the model, so an independent correlation structure for GEE will often be the most appropriate choice^1^. However, in this study, since individuals may participate in more than two survey rounds, contributing more than one observation of change between two surveys, there may be intra-individual correlation between changes over more than two surveys, so an exchangeable correlation structure was defined. This correlation structure assumes that the same correlation exists between observations regardless of the time that passed between measurements (e.g. $Y_{t}$ is correlated with $Y_{t-1}$ in the same way as with $Y_{t-2}$). This is appropriate because few participants participated in more than three surveys. A more complex correlation structure that estimates different correlation coefficients depending on the time that passed between surveys, giving less weight to older measurements, would add little to the model, and estimating more correlation coefficients would lead to a loss of statistical power.

In this study, a change in condom use ($\Delta Condom$) in an individual iover a time period $\Delta t$ is modelled to be dependent on a change in risk perception ($\Delta Riskperception$) in the same individual and same time period:

|  | $\Delta{Condom}_{i\Delta t}=\beta_{0}+\beta_{1}\Delta{Riskperception}_{i\Delta t}+\beta_{Z}{\Delta X}_{i\Delta t}$ | (2) |
| --- | --- | --- |

Where ${\Delta X}_{{it}_{1,2}}$ is a vector of all change variables for individual $i$ in the time period $\Delta t$.

1. Twisk JWR. *Applied Longitudinal Data Analysis for Epidemiology: A Practical Guide*. Cambridge: Cambridge University Press; 2003.

## Preliminary analyses to identify potential confounding factors

Socio-demographic and behavioural characteristics that could confound the relationship between changes in risk perception and condom use were tested for association with both risk perception and condom use. Each variable was tested separately in a logistic generalising estimating equations model for each of these outcomes (Table S3), controlling for age, sex, survey round, and study site. Of the socio-demographic characteristics, marital status, education, and religious affiliation show associations with both risk perception and condom use. School enrolment, socio-economic status, and distance to town are not further considered as they show no association with risk perception and condom use, respectively. STD symptoms in the past 12 months shows strong association with risk perception but only weak association with condom use. Both having sexual risk factors and having a partner who has other partner are strongly associated with risk perception and condom use.

It was also analysed whether these association differed by sex (not shown). Generally, the same patterns of associations exist by sex with the exception of HIV testing (both recent and lifetime). For males, there is a negative association with condom use (aOR=0.88 [0.71-1.09], testing in past 3 years), while for females there is a positive association (aOR=1.41 [1.20-1.65]).

| **Table S3:** Socio-demographic and behavioural characteristics in association with HIV risk perception and condom use during last sex, Manicaland, Zimbabwe, 2003-2013. | | | | | | | | |
| --- | --- | --- | --- | --- | --- | --- | --- | --- |
|  | | **Association with risk perception** | | |  | **Association with condom use** | | |
|  | | **aOR** | **95% CI** | **p-value** |  | **aOR** | **95% CI** | **p-value** |
| Marital status | |  |  |  |  |  |  |  |
|  | Never married | 1 (Reference) | |  |  | 1 (Reference) | |  |
|  | Married | 0.80 | (0.66-0.96) | 0.018 |  | 0.06 | (0.05-0.07) | <0.001 |
|  | Separated/divorced | 0.54 | (0.42-0.70) | <0.001 |  | 0.39 | (0.31-0.50) | <0.001 |
|  | Widowed | 0.35 | (0.27-0.45) | <0.001 |  | 0.19 | (0.14-0.26) | <0.001 |
| Education | |  |  |  |  |  |  |  |
|  | None/primary | 1 (Reference) | |  |  | 1 (Reference) | |  |
|  | Secondary/higher | 0.98 | (0.88-1.08) | 0.657 |  | 1.15 | (1.00-1.33) | 0.058 |
| School enrolment | |  |  |  |  |  |  |  |
|  | No | 1 (Reference) | |  |  | 1 (Reference) | |  |
|  | Yes | 1.24 | (0.86-1.79) | 0.240 |  | 1.49 | (1.06-2.11) | 0.023 |
| Wealth index (tertiles) | |  |  |  |  |  |  |  |
|  | Poorest | 1 (Reference) | |  |  | 1 (Reference) | |  |
|  | 2nd poorest | 0.94 | (0.84-1.06) | 0.317 |  | 1.39 | (1.18-1.63) | <0.001 |
|  | 3rd poorest | 1.06 | (0.93-1.20) | 0.392 |  | 1.49 | (1.25-1.77) | <0.001 |
|  | 4th poorest | 1.10 | (0.93-1.30) | 0.252 |  | 1.45 | (1.16-1.82) | 0.001 |
|  | Least poor | 0.60 | (0.39-0.95) | 0.028 |  | 1.82 | (1.15-2.86) | 0.010 |
| Distance to town | |  |  |  |  |  |  |  |
|  | 0-4km | 1 (Reference) | |  |  | 1 (Reference) | |  |
|  | 5-9km | 0.94 | (0.82-1.08) | 0.383 |  | 0.96 | (0.80-1.15) | 0.620 |
|  | 10+ km | 1.11 | (0.99-1.24) | 0.070 |  | 0.96 | (0.83-1.10) | 0.517 |
| Religion | |  |  |  |  |  |  |  |
|  | Christian mission churches | 1 (Reference) | |  |  | 1 (Reference) | |  |
|  | Apostolic churches | 0.95 | (0.86-1.04) | 0.254 |  | 0.76 | (0.66-0.87) | <0.001 |
|  | Traditional | 0.94 | (0.65-1.36) | 0.747 |  | 0.66 | (0.43-1.02) | 0.062 |
|  | Other | 0.94 | (0.84-1.06) | 0.328 |  | 0.84 | (0.71-0.99) | 0.035 |
|  | No religion stated | 1.13 | (0.95-1.34) | 0.167 |  | 0.78 | (0.64-0.94) | 0.009 |
| STD symptoms | |  |  |  |  |  |  |  |
|  | No symptoms | 1 (Reference) | |  |  | 1 (Reference) | |  |
|  | Yes symptoms | 1.94 | (1.68-2.23) | <0.001 |  | 1.11 | (0.90-1.37) | 0.340 |
| HIV testing (ever) | |  |  |  |  |  |  |  |
|  | No | 1 (Reference) | |  |  | 1 (Reference) | |  |
|  | Yes | 0.90 | (0.82-0.99) | 0.029 |  | 1.06 | (0.94-1.20) | 0.350 |
| HIV testing (past 3 years) | |  |  |  |  |  |  |  |
|  | No | 1 (Reference) | |  |  | 1 (Reference) | |  |
|  | Yes | 0.94 | (0.86-1.03) | 0.169 |  | 1.13 | (1.00-1.28) | 0.045 |
| Sexual risk | |  |  |  |  |  |  |  |
|  | No risk factor | 1 (Reference) | |  |  | 1 (Reference) | |  |
|  | 1 risk factor | 1.43 | (1.24-1.65) | <0.001 |  | 2.91 | (2.52-3.36) | <0.001 |
|  | 2+ risk factors | 2.39 | (1.97-2.90) | <0.001 |  | 3.91 | (3.27-4.67) | <0.001 |
| Partner has other partners | |  |  |  |  |  |  |  |
|  | No | 1 (Reference) | |  |  | 1 (Reference) | |  |
|  | Yes | 1.94 | (1.73-2.16) | <0.001 |  | 2.33 | (2.01-2.71) | <0.001 |
| Values are adjusted odds ratios (aOR), 95% confidence intervals (95% CI), and p-values. Each variable was tested in a separate model, controlling for age, sex, survey round, and study site (covariate results not shown). | | | | | | | | |

## Description of modelling change for potential confounding factors

For the identified potential confounding factors (age, marital status, school enrolment, education, socio-economic status, having symptoms of STDs, HIV testing, sexual risk behaviour, and having a partner who has other partners), change variables were created as outlined in Table S4.

| **Table S4:** Additional change variables included in the analysis. |
| --- |
| **Change in 5-year age-group**  A variable was created to capture no change in five-year age-group and increase into a specific five-year age-group between 20-24 and 50-54 years. This approach was chosen as dummy variables would not create equivalent change variables (participants cannot become members of the lowest age-group of 15-19 years and cannot leave the oldest age-group of 50-54 years).  **Change in marital status**  Similar to change in age-group, dummy variables for marital status would not create equivalent change variables as participants cannot become members of the never married category. A variable was created to capture no change in marital status, becoming married after having never been married before, becoming married after having been divorced or widowed, becoming divorced or widowed after having been married before, and becoming divorced or widowed after having never been married before (marriage is assumed to have happened between surveys). Divorced and widowed statuses were combined due to small sample sizes in each category.  **Change in school enrolment status**  A variable was created to capture no change in school enrolment status, becoming enrolled in school, and leaving school after being previously enrolled.  **Change in education**  A variable was created to capture no change in educational attainment or a change from no/primary education to secondary/higher education.  **Change in socio-economic status (wealth index quintile)**  Dummy variables were created for each wealth index quintile. Change variables were created for each of these with three possible values (no change, joining category, leaving category). Four of the five change variables were included in regression models.  **Change in sexually transmitted disease (STD) symptoms**  A variable was created to capture no change in reporting of STD symptoms, reporting of STD symptoms after reporting no symptoms previously, and reporting no STD symptoms after reporting symptoms before.  **Ever HIV testing**  A variable was created to capture changes in having ever been tested for HIV. A change in this variable indicates that the participant had an HIV test for the first time between surveys.  **Recent HIV testing**  No separate variable was created because the survey measure referred to HIV testing in the past three years, which reflects the experience of HIV testing between surveys (which were about 3 years apart).  **Change in sexual risk**  A sexual risk variable was created based on reporting of multiple partners in the past 12 months, at least one casual partner in the past three years, and more than one sexual partner at the moment, to identify individuals with no, one, or at least two risk factors. Dummy variables were created for each category of sexual risk, and change variables were created for each of these with three possible values (no change, joining category, leaving category). Two of the three change variables were included in regression models.  **Change in perceived partner risk**  Perceived partner risk was based on reporting that the partner has other partners. A variable was created to capture no change in perceived partner risk, reporting that the partner has other partners after not reporting this previously, and reporting that the partner does not have other partners after previously reporting partner risk. |
| Note: Details on data and measures are provided in the supplementary material above. |

##

## Analysis of decrease in condom use

In the main analysis, only increase in condom use was considered by restricting the sample to those who did not report condom use at the beginning of the period between surveys. Decrease in condom use was also considered by restricting the sample to those reporting condom use at the beginning of the period between surveys. A decrease was defined as reporting condom use at the beginning of the period between surveys but not reporting condom use at the end of the period.

*Hypotheses*

In addition to the two hypotheses considered in the main analysis, there are further hypotheses regarding the associations between decreasing condom use and risk perception, as outlined in Table S5.

*Results: Effects of changes in risk perception among those using condoms*

Given low levels of condom use in the study population, sample sizes for those reporting condom use at the beginning of the period between surveys were small (Table S6). Among males, decrease in condom use was less common among those who increased risk perception compared no change or decrease in risk perception (Figure S1), and increased risk perception was weakly negatively associated with decreased condom use (aOR=0.79 [0.41-1.51]) while there was no association for decreased risk perception (aOR=0.92 [0.48-1.78]) (model 2, Table S6). For females, decrease in condom was more common among those who increased risk perception (Figure S1) but the association was weak (aOR=0.86 [0.47-1.56]). A decrease in risk perception was weakly negatively associated with decreased condom use (aOR=0.72 [0.44-1.20]) (model 2, Table S6). Sample sizes were very small when dividing changes in risk perception by reason for risk perception, creating large uncertainty around estimates (Table S6). Nearly no decrease in condom use can be attributed to increased risk perception among either sex (Table S7) and to decreased risk perception among males. 2.97% more cases of decreased condom use would have occurred in the absence of decreased risk perception among females (Table S7).

*Discussion*

Among those reporting condom use at the beginning of periods between surveys, there is no support for the hypotheses that decreased condom use leads to increased risk perception (hypothesis 3(a)) or decreased risk perception leads to decreased condom use (hypothesis 4). However, there is some support for hypothesis 3(b) as males who increased risk perception were less likely to decrease condom use. This further supports the role of risk perception in motivating preventative behaviour. Nevertheless, sample sizes were small for decreases in condom use, so there was limited power to make inferences about associations with changing risk perception.

| **Change in risk perception** |
| --- |

**Figure S1.** Decrease in condom use during last sexual intercourse between surveys among participants with different patterns of change in perceiving a risk for HIV infection (no change; increase; decrease) among HIV-negative, sexually active males and females (15-54 years), Manicaland, Zimbabwe, 2003-2013.

| **Table S5:** Key hypotheses of associations between condom use and risk perception. |
| --- |
| **Sample: Those reporting condom use at the beginning of the period between surveys** |
| *Among those not perceiving a risk for HIV infection at the beginning of the period between surveys:*  Hypothesis 3(a): A decrease in condom use leads to an increase in risk perception  Hypothesis 3b): A decrease in condom use is not or negatively associated with an increase in risk perception  These competing hypotheses are similarly plausible. Regarding hypothesis 3(a), individuals may adjust their risk perception upward when stopping to use condoms between surveys as the individual does no longer engage in the protective behaviour. In this situation, it is implausible that an increase in risk perception causes a decrease in condom use. However, hypothesis 3(b) is also plausible because individuals may stop using condoms when a ‘threat’ is removed and because of the removal of the threat the individuals have no reasons to perceive a risk. Therefore, there may be no or a negative association, so individuals are less likely to increase risk perception when decreasing condom use. Similarly, given that an increase in risk perception should further motivate to engage in preventative behaviour, so it should be ‘protective’ against decreases in condom use (negative association). |
| *Among those perceiving a risk for HIV infection at the beginning of the period between surveys:*  Hypothesis 4: A decrease in risk perception leads to a decrease in condom use  A positive association between decreased risk perception and decreased condom is expected as a decrease in risk perception removes the motivation to engage in protective behaviour, so individuals may stop using condoms. It is implausible that a decrease in condom use causes a decrease in risk perception. |

| **Table S6:** Associations between changes in risk perception and decrease in condom use between surveys, Manicaland, Zimbabwe, 2003-2013. | | | | | | | | | | | |
| --- | --- | --- | --- | --- | --- | --- | --- | --- | --- | --- | --- |
|  | | **Males** | | | | | **Females** | | | | |
| Outcome: Decrease in condom use (vs. no change) | |  | **Model 1**  **(N=594)** | | **Model 2**  **(N=580)** | |  | **Model 1**  **(N=554)** | | **Model 2**  **(N=522)** | |
| Variable | | **n (%)** | **aOR** | **(95% CI)** | **aOR** | **(95% CI)** | **n (%)** | **aOR** | **(95% CI)** | **aOR** | **(95% CI)** |
| Change in risk perception | |  |  | |  | |  |  | |  |  |
|  | No change in risk perception | 472 (79.5) | 1 (Reference) | | 1 (Reference) | | 340 (61.4) | 1 (Reference) | | 1 (Reference) | |
|  | No risk perception → Risk perception (increase) | 57 (9.60) | 0.75 | (0.43-1.31) | 0.79 | (0.41-1.51) | 91 (16.4) | 1.02 | (0.60-1.73) | 0.86 | (0.47-1.56) |
|  | Risk perception → No risk perception (decrease) | 65 (10.9) | 1.13 | (0.66-1.95) | 0.89 | (0.46-1.72) | 123 (22.2) | 0.74 | (0.47-1.14) | 0.72 | (0.44-1.20) |

| Change in risk perception with reason^a^ | | |  |  |  |  |  |  |  |  |  |  |
| --- | --- | --- | --- | --- | --- | --- | --- | --- | --- | --- | --- | --- |
|  | No change in risk perception | | 472 (79.7) | 1 (Reference) | | 1 (Reference) | | 340 (61.9) | 1 (Reference) | | 1 (Reference) | |
|  | No risk perception → Risk perception (reason) | |  |  |  |  |  |  |  |  |  |  |
|  |  | Has multiple partners | 13 (2.20) | 1.64 | (0.51-5.26) | 2.63 | (0.69-10.0) | 6 (1.09) | 0.19 | (0.05-0.77) | 0.17 | (0.03-1.02) |
|  |  | Partner has other partners | 10 (1.69) | 1.38 | (0.39-4.88) | 0.98 | (0.31-3.05) | 23 (4.19) | 0.83 | (0.31-2.25) | 0.71 | (0.21-2.37) |
|  |  | Marry HIV-positive partner | 13 (2.20) | 0.30 | (0.08-1.11) | 0.39 | (0.09-1.74) | 5 (0.91) | 0.89 | (0.14-5.54) | 1.03 | (0.19-5.72) |
|  |  | Other | 20 (3.38) | 0.59 | (0.24-1.44) | 0.44 | (0.15-1.27) | 57 (10.4) | 1.35 | (0.69-2.64) | 1.09 | (0.52-2.27) |
|  | Risk perception (reason) → No risk perception | |  |  |  |  |  |  |  |  |  |  |
|  |  | Has multiple partners | 5 (0.84) | 1.32 | (0.19-9.31) | 0.45 | (0.09-2.18) | 4 (0.73) | 0.28 | (0.06-1.26) | 0.15 | (0.02-0.94) |
|  |  | Partner has other partners | 14 (2.36) | 1.59 | (0.51-5.01) | 1.26 | (0.31-5.08) | 46 (8.38) | 0.67 | (0.36-1.27) | 0.69 | (0.32-1.47) |
|  |  | Marry HIV-positive partner | 33 (5.57) | 0.96 | (0.46-1.98) | 0.83 | (0.34-2.02) | 9 (1.64) | 0.18 | (0.03-1.02) | 0.15 | (0.02-1.02) |
|  |  | Other | 12 (2.03) | 1.38 | (0.41-4.67) | 1.22 | (0.32-4.70) | 59 (10.8) | 0.97 | (0.52-1.79) | 1.03 | (0.53-2.03) |
| Values are: Sample sizes (n) and percentages (%) for changes in risk perception; sample sizes for regression models (N); and adjusted odds ratios (aOR) with 95% confidence intervals (95% CI). Sample: Sexually active, HIV-negative participants (15-54 years) reporting condom use at the beginning of periods between surveys. Outcome of regressions: Stopping condom use (decrease) vs. no change (continuing using condoms). Increased and decreased risk perception was compared to no change (risk perception or no risk perception in both surveys). Estimates for other independent variables are not shown. Sample sizes differ between models due to missing data on included variables.  Model 1: Change variables included for: Age group  Model 2: Change variables included for: Age group, marital status, educational attainment, school enrolment status, socio-economic status, STD symptoms, sexual risk, partner concurrency, HIV testing (lifetime), HIV testing (past three years)  ^a^ Reasons for risk perception refer to the reasons given at the end of the period between surveys for increasing risk perception or at the beginning for decreasing risk perception. | | | | | | | | | | | | |

| **Table S7:** Population attributable fractions for decrease in condoms due to changes in risk perception, Manicaland, Zimbabwe, 2003-13. | | | | | | |
| --- | --- | --- | --- | --- | --- | --- |
|  | **Decrease in condom use** | | | | | |
|  | **Males** | | | **Females** | | |
|  | **n/N (%)** | **PAF** | **(95% CI)** | **n/N (%)** | **PAF** | **(95% CI)** |
| Increased risk perception | 25/308 (8.12) | -0.89% | (-3.31-1.48%) | 58/319 (18.2) | -1.03% | (-5.28-3.04%) |
| Decreased risk perception | 36/308 (11.7) | -0.42% | (-2.87-1.97%) | 66/319 (20.7) | -2.97% | (-7.77-15.9%) |
| Values are: Number of people who increased or decreased risk perception (n) and their percentage (%) among everyone who decreased condom use (N); and population attributable fraction (PAF) and 95% confidence interval (95% CI), indicating the proportion of change in condom use due to the change in risk perception. These estimates are based on adjusted odds ratios (model 2 estimates in Tables S7). | | | | | | |

## Sensitivity analysis: Different definition of ‘no change’ in the change in risk perception variable

In the main analysis, the change in risk perception variable consisted of three categories: No change between surveys (either perceiving a risk or not perceiving a risk in both surveys), increase in risk perception, and decrease in risk perception. This means that, in the regression analyses, increased risk perception was not just compared to those who continued to not perceive a risk and decreased risk perception was not just compared to those who continue to perceive a risk, but in both cases the no change category includes both types of risk perception. In this sensitivity analysis, the regression analyses were conducted comparing increased risk with continuing to not perceive a risk only, so excluding those who perceived a risk in both surveys. A similar analysis could not be implemented for decreased risk perception due to small sample sizes. As can be seen in Tables S8-9, the results in this analysis are similar to those in Table 4 in the main analysis, so conclusions were not affected by the definition of no change in risk perception.

| **Table S8:** Associations between changes in risk perception and increase in condom use between surveys (sensitivity analysis), Manicaland, Zimbabwe, 2003-2013. | | | | | | | | | | | |
| --- | --- | --- | --- | --- | --- | --- | --- | --- | --- | --- | --- |
|  | | **Males** | | | | | **Females** | | | | |
| Outcome: Increase in condom use (vs. no change) | |  | **Model 1 (n=1958)** | | **Model 2 (n=1933)** | |  | **Model 1 (n=2603)** | | **Model 2 (n=2525)** | |
| Variable | | **N (%)** | **aOR** | **(95% CI)** | **aOR** | **(95% CI)** | **N (%)** | **aOR** | **(95% CI)** | **aOR** | **(95% CI)** |
| Change in risk perception | |  |  | |  | |  |  | |  |  |
|  | No change in risk perception | 1752 (89.5) | 1 (Reference) | | 1 (Reference) | | 1781 (68.4) | 1 (Reference) | | 1 (Reference) | |
|  | No risk perception → Risk perception (increase) | 206 (10.5) | 1.85 | (1.20-2.88) | 1.42 | (0.86-2.33) | 822 (31.6) | 1.35 | (1.01-1.81) | 1.21 | (0.88-1.66) |
| Values are: Sample sizes (n) and percentages (%) for changes in risk perception; sample sizes for regression models (N); and adjusted odds ratios (aOR) with 95% confidence intervals (95% CI). Sample: Sexually active, HIV-negative participants (15-54 years) not reporting condom use at the beginning of periods between surveys. Outcome of regressions: Adoption of condom use (increase) vs. no change (continuing not using condoms). Increased and decreased risk perception was compared to no change (risk perception or no risk perception in both surveys). Estimates for other independent variables are not shown. Sample sizes differ between models due to missing data on included variables.  Model 1: Change variables included for: Age group  Model 2: Change variables included for: Age group, marital status, educational attainment, school enrolment status, socio-economic status, STD symptoms, sexual risk, partner concurrency, HIV testing (lifetime), HIV testing (past three years) | | | | | | | | | | | |

| **Table S9:** Associations between changes in risk perception and decrease in condom use between surveys (sensitivity analysis), Manicaland, Zimbabwe, 2003-2013. | | | | | | | | | | | |
| --- | --- | --- | --- | --- | --- | --- | --- | --- | --- | --- | --- |
|  | | **Males** | | | | | **Females** | | | | |
| Outcome: Decrease in condom use (vs. no change) | |  | **Model 1 (n=487)** | | **Model 2 (n=482)** | |  | **Model 1 (n=291)** | | **Model 2 (n=277)** | |
| Variable | | **N (%)** | **aOR** | **(95% CI)** | **aOR** | **(95% CI)** | **N (%)** | **aOR** | **(95% CI)** | **aOR** | **(95% CI)** |
| Change in risk perception | |  |  | |  | |  |  | |  |  |
|  | No change in risk perception | 430 (88.3) | 1 (Reference) | | 1 (Reference) | | 200 (68.7) | 1 (Reference) | | 1 (Reference) | |
|  | No risk perception → Risk perception (increase) | 57 (11.7) | 0.71 | (0.40-1.26) | 0.73 | (0.38-1.39) | 91 (31.3) | 1.05 | (0.61-1.79) | 0.98 | (0.51-1.91) |
| Values are: Sample sizes (n) and percentages (%) for changes in risk perception; sample sizes for regression models (N); and adjusted odds ratios (aOR) with 95% confidence intervals (95% CI). Sample: Sexually active, HIV-negative participants (15-54 years) reporting condom use at the beginning of periods between surveys. Outcome of regressions: Stopping condom use (decrease) vs. no change (continuing using condoms). Increased and decreased risk perception was compared to no change (risk perception or no risk perception in both surveys). Estimates for other independent variables are not shown. Sample sizes differ between models due to missing data on included variables.  Model 1: Change variables included for: Age group  Model 2: Change variables included for: Age group, marital status, educational attainment, school enrolment status, socio-economic status, STD symptoms, sexual risk, partner concurrency, HIV testing (lifetime), HIV testing (past three years) | | | | | | | | | | | |

## Additional results: Associations by age group and marital status

For associations between changes in risk perception and increase in condom use between surveys, Table S10 shows the results for models 1 (controlling for change in age group) by age group (15-24 vs. 25-54 years) for both sexes combined; Table S12 shows these by sex. Generally, both an increase and decrease in risk perception were associated with higher odds of increasing condom use among both younger and older people, with similar effect sizes.

Table S11 shows these models implemented by marital status (controlling for change in age group and change in marital status); Table S13 shows these by sex. While both increase and decrease in risk perception was positively associated with increase in condom use regardless of marital status, the associations were markedly stronger among those not currently married. This particularly true for females (Table S13).

| **Table S10:** Associations between changes in risk perception and increase in condom use between surveys by age groups, Manicaland, Zimbabwe, 2003-2013. | | | | | | | |
| --- | --- | --- | --- | --- | --- | --- | --- |
|  | | **Both sexes combined** | | | | | |
| Outcome: Increase in condom use (vs. no change) | | **15-24 years (N=1253)** | | | **25-54 years (N=6025)** | | |
| Variable | | **n (%)** | **aOR** | **(95% CI)** | **n (%)** | **aOR** | **(95% CI)** |
| Change in risk perception | |  |  |  |  |  |  |
|  | No change in risk perception | 833 (66.5) | 1 (Reference) | | 4252 (70.6) | 1 (Reference) | |
|  | No risk perception → Risk perception (increase) | 232 (18.5) | 1.43 | (0.85-2.39) | 796 (13.2) | 1.53 | (1.18-1.97) |
|  | Risk perception → No risk perception (decrease) | 188 (15.0) | 1.55 | (0.89-2.69) | 977 (16.2) | 1.33 | (1.03-1.71) |
| Values are: Sample sizes (n) and percentages (%) for changes in risk perception; sample sizes for regression models (N); and adjusted odds ratios (aOR) with 95% confidence intervals (95% CI). Sample: Sexually active, HIV-negative participants not reporting condom use at the beginning of periods between surveys. The sample was divided into those aged 15-24 and 25-54 years at the beginning of the period between surveys. Outcome of regressions: Adoption of condom use (increase) vs. no change (continuing not using condoms). Increased and decreased risk perception was compared to no change (risk perception or no risk perception in both surveys). Estimates for other independent variables are not shown. Sample sizes differ between models due to missing data on included variables. Models included change in age group (by five-year age groups) and sex. | | | | | | | |

| **Table S11:** Associations between changes in risk perception and increase in condom use between surveys by marital status, Manicaland, Zimbabwe, 2003-2013. | | | | | | | |
| --- | --- | --- | --- | --- | --- | --- | --- |
|  | | **Both sexes combined** | | | | | |
| Outcome: Increase in condom use (vs. no change) | | **Not currently married (N=1111)** | | | **Currently married (N=6167)** | | |
| Variable | | **n (%)** | **aOR** | **(95% CI)** | **n (%)** | **aOR** | **(95% CI)** |
| Change in risk perception | |  |  |  |  |  |  |
|  | No change in risk perception | 761 (68.5) | 1 (Reference) | | 4324 (70.1) | 1 (Reference) | |
|  | No risk perception → Risk perception (increase) | 158 (14.2) | 1.86 | (1.23-2.83) | 870 (14.1) | 1.38 | (1.02-1.87) |
|  | Risk perception → No risk perception (decrease) | 192 (17.3) | 1.60 | (1.10-2.33) | 973 (15.8) | 1.13 | (0.82-1.54) |
| Values are: Sample sizes (n) and percentages (%) for changes in risk perception; sample sizes for regression models (N); and adjusted odds ratios (aOR) with 95% confidence intervals (95% CI). Sample: Sexually active, HIV-negative participants not reporting condom use at the beginning of periods between surveys. The sample was divided into those not currently married (never married, widowed, divorced) and currently at the beginning of the period between surveys. Outcome of regressions: Adoption of condom use (increase) vs. no change (continuing not using condoms). Increased and decreased risk perception was compared to no change (risk perception or no risk perception in both surveys). Estimates for other independent variables are not shown. Sample sizes differ between models due to missing data on included variables. Models included change in age group (by five-year age groups) and in marital status as well as sex. | | | | | | | |

| **Table S12:** Associations between changes in risk perception and increase in condom use between surveys by sex and age group, Manicaland, Zimbabwe, 2003-2013. | | | | | | | | | | | | | |
| --- | --- | --- | --- | --- | --- | --- | --- | --- | --- | --- | --- | --- | --- |
|  | | **Males** | | | | | | **Females** | | | | | |
| Outcome: Increase in condom use (vs. no change) | | **15-24 years (N=264)** | | | **25-54 years (N=1930)** | | | **15-24 years (N=989)** | | | **25-54 years (N=4095)** | | |
| Variable | | **n (%)** | **aOR** | **(95% CI)** | **n (%)** | **aOR** | **(95% CI)** | **n (%)** | **aOR** | **(95% CI)** | **n (%)** | **aOR** | **(95% CI)** |
| Change in risk perception | |  |  |  |  |  |  |  |  |  |  |  |  |
|  | No change in risk perception | 210 (79.6) | 1 (Reference) | | 1602 (83.0) | 1 (Reference) | | 623 (63.0) | 1 (Reference) | | 2650 (64.7) | 1 (Reference) | |
|  | No risk perception → Risk perception (increase) | 30 (11.4) | 1.89 | (0.78-4.54) | 176 (9.12) | 1.76 | (1.07-2.88) | 202 (20.4) | 1.38 | (0.73-2.64) | 620 (15.1) | 1.45 | (1.08-1.96) |
|  | Risk perception → No risk perception (decrease) | 24 (9.09) | 1.64 | (0.68-3.98) | 152 (7.88) | 1.95 | (1.17-3.24) | 164 (16.6) | 1.42 | (0.71-2.84) | 825 (20.2) | 1.19 | (0.90-1.58) |
| Values are: Sample sizes (n) and percentages (%) for changes in risk perception; sample sizes for regression models (N); and adjusted odds ratios (aOR) with 95% confidence intervals (95% CI). Sample: Sexually active, HIV-negative participants not reporting condom use at the beginning of periods between surveys. The sample was divided into those aged 15-24 and 25-54 years at the beginning of the period between surveys. Outcome of regressions: Adoption of condom use (increase) vs. no change (continuing not using condoms). Increased and decreased risk perception was compared to no change (risk perception or no risk perception in both surveys). Estimates for other independent variables are not shown. Sample sizes differ between models due to missing data on included variables. Models included change in age group (by five-year age groups). | | | | | | | | | | | | | |

| **Table S13:** Associations between changes in risk perception and increase in condom use between surveys by sex and marital status, Manicaland, Zimbabwe, 2003-2013. | | | | | | | | | | | | | |
| --- | --- | --- | --- | --- | --- | --- | --- | --- | --- | --- | --- | --- | --- |
|  | | **Males** | | | | | | **Females** | | | | | |
| Outcome: Increase in condom use (vs. no change) | | **Not currently married (N=257)** | | | **Currently married (N=1937)** | | | **Not currently married (N=854)** | | | **Currently married (N=4230)** | | |
| Variable | | **n (%)** | **aOR** | **(95% CI)** | **n (%)** | **aOR** | **(95% CI)** | **n (%)** | **aOR** | **(95% CI)** | **n (%)** | **aOR** | **(95% CI)** |
| Change in risk perception | |  |  |  |  |  |  |  |  |  |  |  |  |
|  | No change in risk perception | 206 (80.2) | 1 (Reference) | | 1606 (82.9) | 1 (Reference) | | 555 (65.0) | 1 (Reference) | | 2718 (64.3) | 1 (Reference) | |
|  | No risk perception → Risk perception (increase) | 19 (7.39) | 2.34 | (0.87-6.29) | 187 (9.65) | 1.78 | (1.03-3.08) | 139 (16.3) | 1.94 | (1.24-3.03) | 683 (16.2) | 1.18 | (0.82-1.71) |
|  | Risk perception → No risk perception (decrease) | 32 (12.5) | 1.52 | (0.76-3.03) | 144 (7.43) | 1.69 | (0.92-3.14) | 160 (18.7) | 1.63 | (1.05-2.54) | 829 (19.6) | 1.02 | (0.72-1.44) |
| Values are: Sample sizes (n) and percentages (%) for changes in risk perception; sample sizes for regression models (N); and adjusted odds ratios (aOR) with 95% confidence intervals (95% CI). Sample: Sexually active, HIV-negative participants not reporting condom use at the beginning of periods between surveys. The sample was divided into those not currently married (never married, widowed, divorced) and currently at the beginning of the period between surveys. Outcome of regressions: Adoption of condom use (increase) vs. no change (continuing not using condoms). Increased and decreased risk perception was compared to no change (risk perception or no risk perception in both surveys). Estimates for other independent variables are not shown. Sample sizes differ between models due to missing data on included variables. Models included change in age group (by five-year age groups) and in marital status. | | | | | | | | | | | | | |
